# Technical note: MRI lymphangiography of the lower limb in secondary lymphedema

**DOI:** 10.4103/0971-3026.76047

**Published:** 2011

**Authors:** Ravindra B Kamble, Roshan Shetty, N Diwakar, G Madhusudan

**Affiliations:** Department of Radiology, BGS Global Hospital, Bangalore, India; 1 Department of Plastic Surgery, BGS Global Hospital, Bangalore, India

**Keywords:** Gadopentetate, lymphangiography, lymphedema, MRI

## Abstract

We would like to describe a case of MRI lymphangiography of the left leg, performed by subcutaneous injection of gadopentetate in the foot, followed by serial acquisitions of images, in a 52-year-old female, who presented to us with progressive leg swelling following total hysterectomy and radiation therapy. Successful demonstration of lymphatic channels, along with faint visualization of the venous system, was achieved. This technique allows excellent visualization of lymphatic channels.

## Introduction

The credit for the first description of lymphatic channels goes to the Italian anatomist Gasparo Asellius who saw these milky white channels in a dog.[1] However, Kinmonth *et al*. were the first to demonstrate lymphatics after injecting a blue dye subcutaneously.[2] Since then, there have been various advances in the imaging techniques for identifying and visualizing the lymphatic system and its diseases. Techniques described in the past include lymphangiography and lymphoscintigraphy.[3,4] We describe an MRI lymphangiography (MRL) technique in a case of secondary lymphedema following surgery and radiation for endometrial carcinoma.

## Case Report

A 52-year-old lady presented to us with progressive, huge, swelling of her left leg of 5 year’s duration and swelling of her right leg for the past 1 year. She had been diagnosed to have endometrial carcinoma in 2002 and had undergone radical hysterectomy and pelvic lymph node dissection, followed by 35 fractions of radiotherapy. She had been asymptomatic till 2005.

Doppler study for both legs showed a normal venous system. MRI angiography of both legs showed a normal arterial system. A CT scan of the abdomen done 7 months back had shown no tumor recurrence or metastases.

**Figure 1 (A-D):**
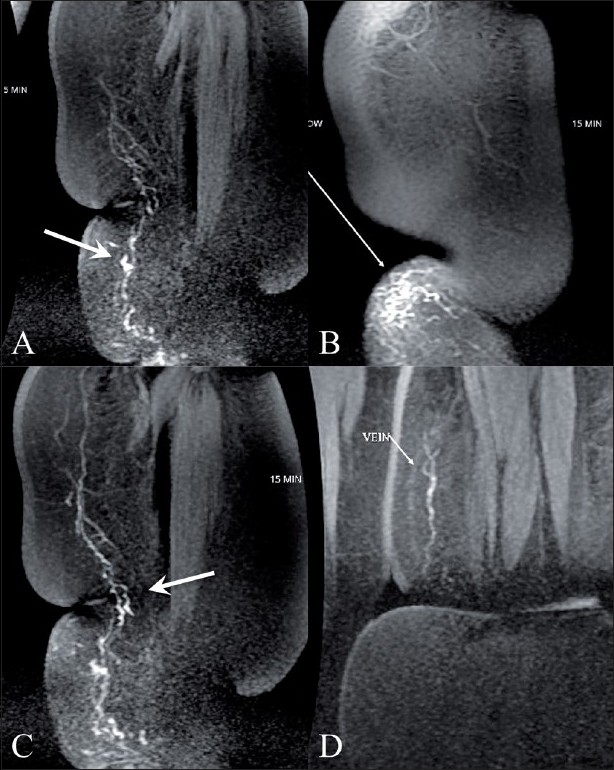
3D spoiled gradient-echo MRI images obtained at 5 min (A,B), 15 min (C) and 35 min (D) shows the lymphatics (arrows in A,C) with dermal backflow (arrow in B) and faint visualization of the veins at 35 min (arrow in D)

**Figure 2:**
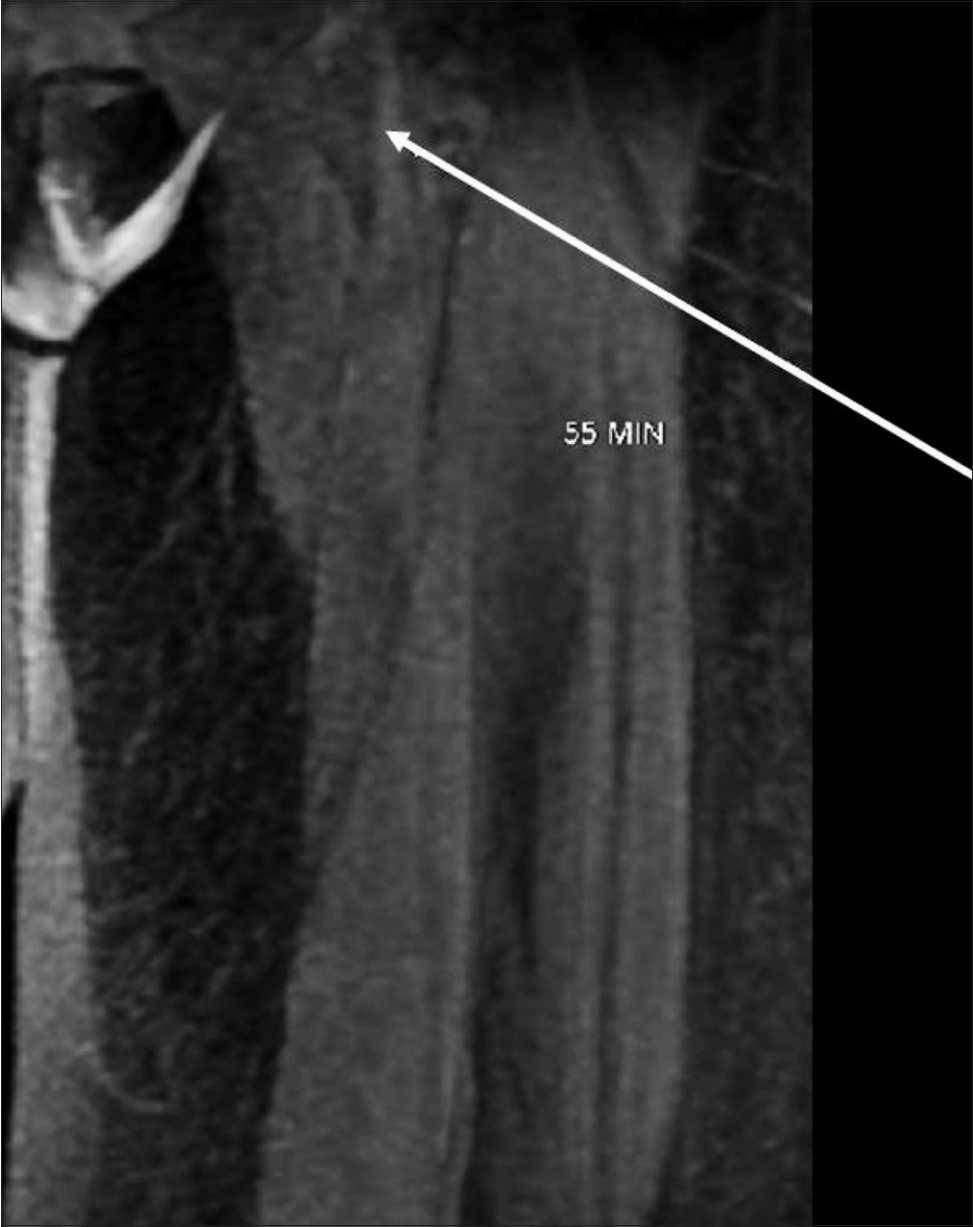
3D spoiled gradient-echo MRI image obtained at 55 min shows dilated lymphatics (arrow) reaching upto the groin

MRL of the left leg was performed on a 1.5-T (Wipro GE, Milwaukee, WI, USA) machine after obtaining informed consent. Following painting and draping of her left foot, 0.5 ml of 2% lignocaine was injected subcutaneously into the interdigital web spaces and between the first and second proximal metatarsal space with a 24-G needle. Following this, 1 ml of meglumine gadopentetate (Magnevist, Bayer Schering Pharma, Berlin, Germany) was injected into each of these five sites at the recommended dose (for intravenous use) of 0.1 mmol/kg body weight. The injected sites were massaged for 1 min. Imaging was performed using a 3D spoiled gradient-echo sequence (LAVA-XV) with the following parameters (TR-4, TE-1.9, TI-7, bandwidth 62.5 kHz, matrix 320 × 192, NEX-0.73, thickness 4 mm with 0 interslice gap, FOV 48 × 43.2, and scan time of 28 s). The scan was performed with an eight-channel body array coil. The acquisition was done at 5, 15, 25, 35, 45, and 55 min [Figure 1]. Dilated lymphatic channels were first visualized at 5 min [Figure 1A] followed by 15 min [Figure 1C], 35 min [Figure 1D] and by 55 min, lymphatics up to the groin could be seen [Figure 2]. There was excellent visualization of multiple lymphatic channels on the superomedial aspect of the leg and, in addition, there was also faint visualization of the veins. Dermal backflow [Figure 1B] was noted in the medial aspect of the lower leg at the ulcer site. No dilated lymphatics were noted in the lateral and posterior aspects of the leg. The patient was kept on antibiotics after the procedure. There was no pain at the puncture site and no other complications were noted.

## Discussion

The incidence of symptomatic lymphedema in a single lower extremity after surgery for uterine corpus malignancy has been found to be 69%, while bilateral lymphedema occurs in 31% of cases. Lymphedema develops after a median time of 5.3 months after the initial surgery.[5] This development of lower extremity lymphedema is associated with the removal of 10 or more lymph nodes during surgery.

Lymphovenous anastomosis is often performed to treat lymphedema. For this, prior visualisation of the lymphatic channels is important. The more the number of lymphovenous anastomoses performed, the better are the results.

Initially, invasive techniques of the exploration of lymphatic vessels and injection of oil-based dyes were used for the assessment of the lymphatic drainage, but these are no longer used due to technical difficulties, the nonavailability of the contrast medium, and associated risks like pulmonary embolism.[3] Lymphoscintigraphy is another technique that detects peripheral lymphatics and can yield quantitative flow information but has limited use in the evaluation of mild lymphedema. It suffers from poor spatial and temporal resolution.[4]

With the development of new MRI sequences, it is now feasible to demonstrate lymphatic channels with MRL and thus help the surgeons plan adequate surgery.[6] This can be achieved with or without the use of contrast. Laor *et al*. have demonstrated lymphatic channels noninvasively in children and infants with lymphatic pathologies, but in a few cases they were unable to suppress signals from veins.[7] Excellent visualization of dilated lymphatic channels is possible with intracutaneous injection of gadodiamide or gadolinium-DTPA, without much venous contamination.[8,9]

We have modified the technique by using a subcutaneous injection of 1 ml of meglumine gadopentetate in each interdigital web space as well as between the first and second metatarsals after first injecting the sites with 0.5 ml of 2% lignocaine. We were able to clearly demonstrate the lymphatic channels and also faintly visualize the venous system.
